# A diagnostic support system based on pain drawings: binary and k-disease classification of EDS, GBS, FSHD, PROMM, and a control group with Pain2D

**DOI:** 10.1186/s13023-023-02663-z

**Published:** 2023-03-28

**Authors:** D. Emmert, N. Szczypien, Tim T. A. Bender, L. Grigull, A. Gass, C. Link, F. Klawonn, R. Conrad, M. Mücke, J. Sellin

**Affiliations:** 1 Center for Rare Diseases Bonn (ZSEB), University Hospital Bonn, Bonn, Germany; 2 Institute for Virology, University Hospital Bonn, Bonn, Germany; 3 Institute for Information Engineering, Ostfalia University of Applied Sciences, Wolfenbüttel, Germany; 4 Biostatistics Group, Helmholtz Centre for Infection Research, Braunschweig, Germany; 5 Clinic for Anesthesiology and Operative Intensive Care Medicine, Department of Pain Medicine, University Hospital Bonn, Bonn, Germany; 6 Department of Psychosomatic Medicine and Psychotherapy, University Hospital Muenster, Muenster, Germany; 7 Institute for Digitalization and General Medicine, University Hospital RWTH Aachen, Aachen, Germany; 8 Center for Rare Diseases Aachen (ZSEA), University Hospital RWTH Aachen, Aachen, Germany

**Keywords:** ORPHA: 269, ORPHA: 606, ORPHA: 2103, ORPHA: 287, Diagnostic support, AI, Rare diseases, k-disease classification, Pain drawings, Machine learning

## Abstract

**Background and objective:**

The diagnosis of rare diseases (RDs) is often challenging due to their rarity, variability and the high number of individual RDs, resulting in a delay in diagnosis with adverse effects for patients and healthcare systems. The development of computer assisted diagnostic decision support systems could help to improve these problems by supporting differential diagnosis and by prompting physicians to initiate the right diagnostic tests. Towards this end, we developed, trained and tested a machine learning model implemented as part of the software called Pain2D to classify four rare diseases (EDS, GBS, FSHD and PROMM), as well as a control group of unspecific chronic pain, from pen-and-paper pain drawings filled in by patients.

**Methods:**

Pain drawings (PDs) were collected from patients suffering from one of the four RDs, or from unspecific chronic pain. The latter PDs were used as an outgroup in order to test how Pain2D handles more common pain causes. A total of 262 (59 EDS, 29 GBS, 35 FSHD, 89 PROMM, 50 unspecific chronic pain) PDs were collected and used to generate disease specific pain profiles. PDs were then classified by Pain2D in a leave-one-out-cross-validation approach.

**Results:**

Pain2D was able to classify the four rare diseases with an accuracy of 61–77% with its binary classifier. EDS, GBS and FSHD were classified correctly by the Pain2D k-disease classifier with sensitivities between 63 and 86% and specificities between 81 and 89%. For PROMM, the k-disease classifier achieved a sensitivity of 51% and specificity of 90%.

**Conclusions:**

Pain2D is a scalable, open-source tool that could potentially be trained for all diseases presenting with pain.

## Introduction and background

Rare diseases pose particular challenges for health care systems as a result of their infrequency, diversity and often complex symptomatology. Especially the diagnosis of rare diseases (RDs) is often difficult, with adverse consequences for affected individuals and health care systems. A disease is categorized as rare in the European Union if it affects less than 1 in 2000 people [1]. Similar definitions exist in other regions of the world (for example, the NIH defines a disease as rare if it affects less than 200,000 people in the US [2]). As there are more than 7000 known individual rare diseases, resulting in an estimated 30 Mio affected in the EU and about 400 Mio people worldwide, it is apparent that the sheer number of possibilities makes it impossible for individual physicians to know all of them. In addition, many rare diseases present with multifaceted clinical symptoms. As a result, affected individuals often wait for a long time until they receive the correct diagnosis (~ 7 years on average [3]). Long time to diagnosis contributes to mental, physical and social distress. In addition, due to many medical consultations and resulting redundant diagnostic procedures, the burden for health care systems is further increased [3].

One possible strategy to improve time to diagnosis for rare diseases is the development of computer assisted diagnostic aids. In recent years, a number of studies and reviews focused on such technical solutions to improve diagnosis of rare diseases ([4–6] and references therein). Clinical decision support systems (CDSSs), while used frequently in clinical settings, are not yet widespread in the context of diagnosis (also referred to as diagnosis decision support systems, DDSSs). Negative physician biases, insufficient accuracy and lacking integration with clinical information systems in use are discussed as underlying the lack of acceptance of DDSSs, in spite of promising results with regard to their effectiveness [7, 8]. This is to the disadvantage of people with rare diseases (RDs), who could benefit from diagnostic aid tools, as diagnosis of RDs is hampered by lack of knowledge and unspecific symptomatology of many RDs [3]. Typically, the “diagnostic odyssey” of patients with undiagnosed rare diseases starts with consultation of family doctors or in other out-patient primary care facilities. In many countries, including Germany, this setting is not ideal for the development of DDSSs, as accessibility and interoperability of healthcare data and information systems is mostly restricted to academic institutions, like university hospitals or big clinics. We and others have therefore concentrated on information that is independent of these systems, like, e.g., patient reported experience measures (PREMs).

For example, questionnaires covering the patient’s history have been successfully used in the past to develop machine learning driven DDSSs. These were developed by analyzing patient interviews and extracting typical experiences before diagnosis to formulate questions. The resulting questionnaires were collected from diagnosed patients, and used successfully to train classifiers, like random forest, support vector machine and neural networks, as well as a combination of those [9–11].

In a different approach, the internationally developed diagnostic aid tool Face2Gene uses portrait photographs and deep learning-based face recognition algorithms for the diagnosis of rare genetic syndromes in children. The underlying facial image analysis framework, DeepGestalt, was trained and curated in an impressive community driven effort [12–14]. It relies on the fact that genetic disorders often present with typical facial features that can be recognized by Face2Gene/DeepGestalt. It has been trained with an impressive ~ 17,000 syndromal portrait images to recognize more than 200 genetic disorders.

Many patients with rare diseases suffer from chronic pain, and it is often the first symptom that leads to medical consultation [15], making pain assessment a promising route towards diagnosis of rare diseases. Pain drawings (PDs) can be understood as an alternative form of PREMs like classical questionnaires, as they are used to communicate the experience of pain from patients to health care providers. Patients mark painful body regions in a simple line drawing of the human body to indicate where they experience pain. This often results in a more precise description of affected body parts than just via verbal description. PDs were first established by Palmer in 1949 [16]. While there are a growing number of studies dealing with the topic of PDs [17–22], none of them focuses on their usefulness as a diagnostic tool for rare diseases. We therefore decided to develop a DDSS based on PDs.

PDs—as image information—are not well suited for standard classifiers, which typically use numerical values, leading to extreme overfitting if every pixel is considered a feature, as the number of features then far exceeds the number of samples. On the other hand, while deep learning with, e.g., convoluted neural networks (CNNs) works well on images, they need large training data sets. These are difficult to obtain for rare diseases, which poses problems for most studies with rare diseases, including clinical trials [23]. Therefore, we searched for alternative approaches and decided to utilize Ružička similarity, which is a suitable measure to calculate similarity between images.

With this study we investigate whether a k-disease classifier based on Ružička similarity is suitable as a DDSS for the detection of rare diseases causing chronic pain in patients. We have previously shown that a binary classifier (part of Pain2D-Tool of the PD analyzing software Pain2D) is able to distinguish between two rare diseases, Ehlers-Danlos syndrome (EDS) and Guillain–Barré syndrome (GBS) [24]. Here, we test the performance of the binary classifier, as well as a new k-disease classifier implemented into Pain2D, on four different diseases (EDS, GBS, facioscapulohumeral muscular dystrophy (FSHD) and proximal myotonic myopathy (PROMM)) and a control group of patients with non-specific chronic pain.

EDS is a group of inherited disorders affecting the connective tissue with a prevalence between 1:150,000 and 1:5000, depending on the population [25]. EDS can present clinically with variable symptomatology, from mild skin hyperextensibility, joint hypermobility, and tissue fragility, to severe physical disability and life-threatening vascular complications [26].

GBS is caused by autoantibodies attacking peripheral nerve components triggered by an infection, resulting in a polyradiculoneuropathy with variable clinical presentation [27]. 1.1 to 1.8 per 100,000 persons suffer from GBS each year [28]. Symptoms can include a range from ascending bilateral limb weakness to decreased reflexes and severe back or extremity pain [29, 30].

FSHD is an autosomal-dominantly inherited muscular dystrophy which characteristically affects facial muscles, shoulder girdles, and upper arms [31]. The prevalence of FSHD is estimated to range between 2.03 and 6.8 per 100,000 individuals [32]. Pain in the affected regions is a common symptom of patients suffering from FSHD [33]. The diagnosis of FSHD can be challenging, especially in milder forms, as typical symptoms of FSHD may not be present [34].

PROMM is a subtype of myotonic dystrophies also referred to as myotonic dystrophy type 2 (DM2). Myotonic dystrophies are autosomal-dominantly inherited diseases that have in common muscular involvement (myotonia, muscle weakness, muscular dystrophy), eye manifestations (early onset cataracts), cardiac conduction defects, and endocrine disorders [35]. Only a few studies deal with the prevalence of PROMM, with estimates for Europe ranging between 9:100,000 [36, 37] and 1:1830 in Finland [38]. As the name implies, PROMM in contrast to DM1 typically affects proximal muscles [39]. 50–80% of PROMM patients suffer from pain, which can be exercise-related, musculoskeletal, or abdominal [40]. While myotonic dystrophies are the most common forms of adult-onset dystrophies, PROMM is likely underdiagnosed due to its heterogeneous phenotype and unclear age of onset [39].

The four diseases and the control group were chosen to cover a range from very different causes of pain (e.g., GBS as an inflammatory disease vs. FSHD as an inherited neuromuscular disease) to more similar causes (e.g., FSHD and PROMM as two autosomal-dominantly inherited neuromuscular diseases) to test the ability of the Pain2D classifiers to distinguish between more or less similar rare diseases. The control group was added to elucidate if more common and unspecific causes of pain can be separated from rare diseases with Pain2D-Tool.

Taken together, our study explores the feasibility of Pain2D for pain-based diagnosis of rare diseases and provides the following contributions:

- Pain2D-generated pain profiles visualize the typical distribution of pain in EDS, GBS, FSHD and PROMM. Pain profiles are based on merging all available pain drawings and provide a color-coded, intuitive overview over the percentages of patients suffering from pain in a given area of the body per disease. Our study thereby provides additional insights about the characteristic pain localizations of the four diseases investigated and the potential to provide such information for other diseases in the future.
- By investigating whether Pain2D could be used as a DDSS, we address the problem of the diagnostic odyssey of patients suffering from rare diseases. As a proof-of-principle study, our results confirm that with the introduction of a k-disease classifier, Pain2D can separate more than two rare diseases (as investigated by Wester et al. [24]) and therefore has the potential to become a useful DDSS for rare disease diagnosis.
- By adding a control group of non-rare causes of pain, this is, to our knowledge, the first time a study investigates whether pain drawings can be used to distinguish rare diseases from common causes of chronic pain.

## Material and methods

### Study design and data collection

Between 2017 and 2019, a total of 35 patients with FSHD (10 male, 25 female) and 90 patients with PROMM participated in this study. Of the latter, one PD was empty and had to be excluded from further analysis, resulting in the inclusion of 89 PROMM PDs (29 male, 60 female participants). Patients were recruited at the neuromuscular out-patient clinic of University Hospital Bonn, Germany. In addition, we contacted the German association for neuromuscular diseases to support us in finding patients willing to participate in our study. Inclusion criteria were a confirmed diagnosis, age above 18 years, and written informed consent.

Between 2019 and 2020, a total of 50 participants with unspecific chronic pain (19 male, 31 female) were recruited for this study (the limiting factor was the number of included participants with rare diseases due to small numbers of patients, so that we adjusted the size of the chronic pain group accordingly.) Inclusion criteria for this group were chronic pain due to a common disease (e.g., post-zoster neuralgia) above 6 months duration and age above 18 years. Exclusion criteria for this group were rare comorbidities. These patients were recruited at the out-patient pain clinic of University Hospital Bonn and at general medicine practices in Bonn, Germany.

In this study, 35 PDs of facioscapulohumeral muscular dystrophy (FSHD), 89 PDs of proximal myotonic dystrophy (PROMM), and 50 PDs from a control group with common causes of chronic pain (CP) were included. Based on genetic findings, two subtypes of FSHD (FSHD1 and FSHD2) can be differentiated, but both have a similar clinical phenotype [31]. We therefore did not distinguish between the two subtypes in our study population. In addition, we used 88 PDs from two different rare diseases that were recruited as part of a previous study: 59 from Ehlers-Danlos syndrome (EDS) and 29 from Guillain Barré syndrome (GBS) [24].

Recruited participants filled in pain drawings as previously described [24].

In accordance with German privacy laws and the declaration of Helsinki, PDs were pseudonymized before sending them from the *Center for Rare Diseases Bonn (ZSEB)* to the analysis server.

This study was registered at the German register for clinical studies DRKS (DRKS-ID: DRKS00014776 (participants recruited for this study) and DRKS00014777 (previously recruited participants, [24]).

### Pain2D: software package for pain drawing analysis

Pain2D is a software package previously developed by our group [24] for the automated processing and analysis of pain drawings based on a template, which can be printed and filled out on paper. As part of the package, an application for tablets (Pain2D-Tablet, paperless) is available, which was however not used for this study. The application Pain2D is open-source and holds a GPL v3.0 license for researchers who want to participate in its future development or test their own pain drawings with the classifiers we have implemented. The application was developed with the open-source statistics and graphics software R and RShiny. For more detailed information about Pain2D, please visit www.pain2d.com.

Pain2D generates pain profiles (PPs, also known as pain frequency maps [19]) by overlapping all PDs which belong to one diagnostic group and computing for each pixel the relative frequency of the PDs in which the pixel was marked. They are depicted as color coded heatmaps, with lower to higher percentages of marked pixels in the summarized PDs labeled in blue to red, with yellow indicating 50% of PDs had marked that pixel. Pixels that were empty in all PDs are depicted in white.

Pain2D offers a binary and a k-disease classifier. The function of the binary classifier has been previously described [24]. Both classifiers first compute a pain profile for each diagnostic group based on the available pain drawings for the corresponding diagnostic group by overlapping all PDs. The classifier assigns a given pain drawing to the disease to which it has the highest Ružička similarity. The Ružička similarity between a pain profile and a pain drawing is defined as $\frac{{\mathop \sum \nolimits_{i = 1}^{m} {\text{min}}\left( {x_{i} , y_{i} } \right)}}{{\mathop \sum \nolimits_{i = 1}^{m} {\text{max}}\left( {x_{i} , y_{i} } \right)}}$ where $m$ is the number of pixels, $x_{i}$ the intensity of pixel $i$ in the pain profile and $y_{i}$ is 1 if the pixel $i$ was marked in the corresponding pain drawing and 0 otherwise.

### Considerations for classification and comparison with other classification methods

Classification based on diagnoses requires a supervised learning method because the target variable (the class) corresponds to the diagnosis (in this case EDS, GBS, FSHD, PROMM and unspecific chronic pain). Standard machine learning or statistical methods like logistic regression, linear discriminant analysis, decision trees, random forests or support vector machines were not considered here as the PDs do not offer a set of features required as input for such classifiers. While it is theoretically possible to consider each pixel as one feature, this would result in too many features compared to the available sample sizes, which are limited due to the rarity of the diseases (in numbers, the result would be 477,400 or 134,270 features, with the latter number resulting from the pixels inside the human body outlines only). The above-mentioned classification methods would therefore require feature selection, which however is also not feasible with such a large number of features and a limited sample size, as too many random correlations would make it impossible to identify the relevant features. A similar problem exists when considering convolutional neural networks (CNNs), which are often applied for image classification: CNNs also require a much larger number of samples per class in the training set than the samples that are available in our data set.

Classification based on Ružička similarity as a suitable measure to compare images was therefore chosen to circumvent these problems. We tested classification based on Ružička similarity with a simple nearest neighbor classifier and with a classifier based on the similarity to the pain profile (see above). The latter method was chosen as it resulted in much better accuracy.

To explore other methods of classification for comparison, we also tested approaches based on kernel nearest neighbor classifiers with different kernels: The standard Gaussian kernel gave the best results but could not reach the performance of the classifier based on pain profiles. Sensitivity for the four disease classes ranged from 0.03 to 0.85 for the Gaussian kernel and—as another example—0.20–0.56 for the inverse kernel. We have also used other kernels, which proofed to perform poorly.

### Statistical evaluation

Because of the limited size of the data set, evaluation was carried out by leave-one-out cross-validation (LOOCV). For the classification results, receiver operating characteristic (ROC) curves were plotted and AUC values calculated using the pROC package from R. For ROC analysis, resulting Ružička similarities to the diagnostic groups were normalized, so that the sum of the normalized Ružička similarities to the diagnostic groups is 1. Confidence intervals were calculated as indicated. The leave-one-out-cross-validated confusion matrices were tested with Fisher’s exact test (binary classification) or *χ*^2^ test (k-disease classification) for better than random classification, as indicated.

## Results

### Pain profiles of the five diagnostic groups (EDS, GBS, PROMM, FSHD, CP)

Pain2D generates pain profiles, which provide a visual result of the sum of all PDs of one diagnostic group and serve as the basis for similarity measurement of an individual PD for classification by Pain2D.

#### EDS

**Fig. 1:**
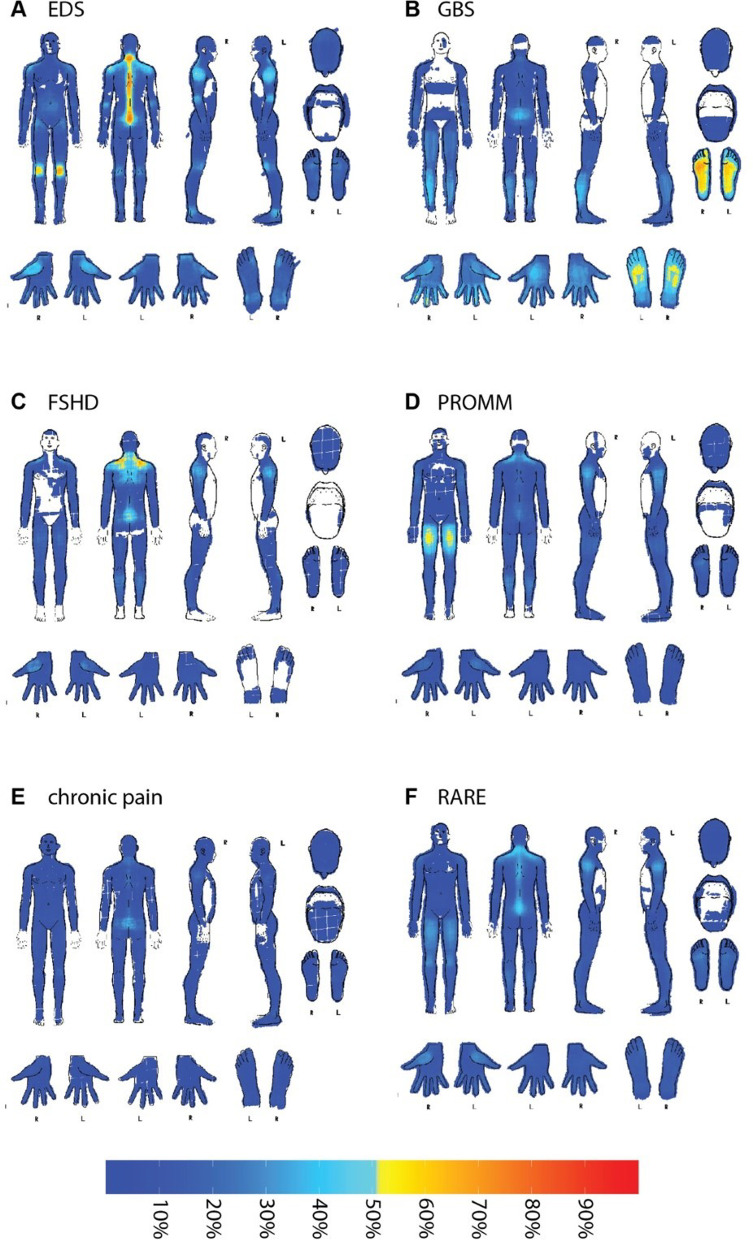
Pain profiles of the five diagnostic groups used in this study, EDS (**A**), GBS (**B**), FSHD (**C**), PROMM (**D**), chronic pain (CP, **E**) and RARE (**F**). The depicted pain profiles were constructed by Pain2D from 29 EDS (**A**), 59 GBS (**B**), 35 FSHD (**C**), 89 PROMM (**D**) and 50 CP (**E**) PDs. RARE is based on 29 EDS, 59 GBS, 35 FSHD and 89 PROMM PDs (**F**)

The pain profile of EDS reveals that most patients experience pain along the vertebral column with the neck and the tailbone, and the knee joints. These regions were marked by approximately 70% of the participating patients. In addition, nearly 50% of the participants marked the shoulder region, the elbows and the thumb saddle joint (Fig. 1A; compare [24]).

#### GBS

The most prominent regions marked by patients were the dorsal and plantar side of the feet (~ 70% of patients). In addition, about 50% marked the palmar side of the fingertips, the dorsal side of the left palm and the tailbone (Fig. 1B; compare [24]).

#### FSHD

As shown in Fig. 1C, the most frequent body regions marked by patients with FSHD are the shoulders and the lower back with percentages of 50–60%. In addition, the upper arms were marked by ca. 40% (Fig. 1C).

#### PROMM

The pain profile of PROMM shows that around 50–60% of patients marked the upper legs as a painful region. Other less frequent regions marked were the shoulders, the lower legs and the lower back (Fig. 1D).

#### Chronic pain (CP)

The disease pattern of our control group (Fig. 1E) shows that pain patterns are more equally distributed between patients, with percentages marked well below 50%. The localization with the highest percentage is the lower back with ca. 40%. This result is consistent with the expectation of a more unspecific pain pattern as this group was suffering from various common causes of chronic pain.

#### RARE

We also generated a pain profile of all four rare diseases (EDS, GBS, PROMM and FSHD) in order to test if PDs from a group of rare diseases show similarities that allow distinction from other causes of pain. Accordingly, the resulting pain profile shows the typical pain areas from all four rare diseases, but at lower percentages (as a sum projection of the four RD pain profiles; Fig. 1F). This “consensus” pain profile is however not equally informed by the four rare diseases, as different numbers of individual PDs for each disease were included. For example, the data set contains 89 PDs of PROMM, but only 29 of GBS.

##### The binary classifier of Pain2D can differentiate between a group of rare diseases (comprised of EDS, GBS, FSHD and PROMM) and unspecific chronic pain (CP) with high sensitivity but lower specificity

**Fig. 2:**
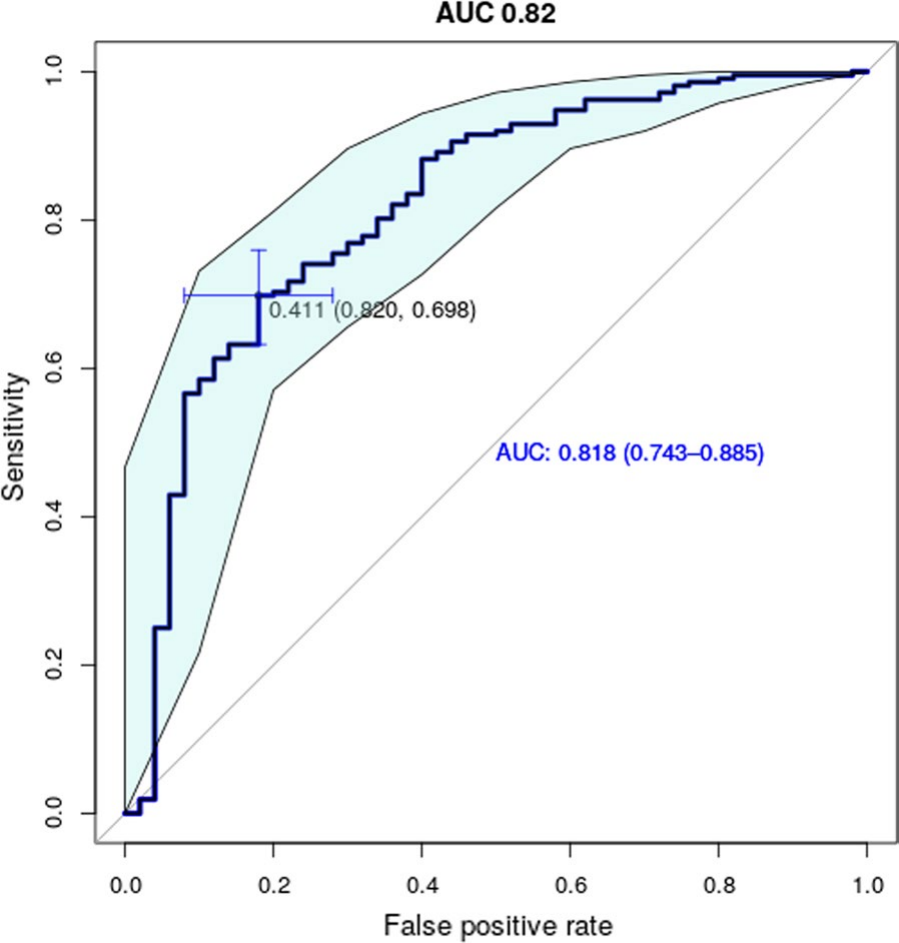
ROC curve for classification of PDs into RARE and CG with the binary classifier of Pain2D. The light blue area indicates the 95% confidence interval. Blue crosshairs indicate optimal classification threshold of 0.41

We were interested if PDs can be used to predict the presence of a rare disease as opposed to a common cause for chronic pain. As a test, we grouped all four rare diseases into the group RARE and classified all PDs (EDS, GBS, FSHD, PROMM, CP) with the binary classifier of Pain2D into RARE or CP. With the standard threshold of 0.5 for the binary classifier we reached a very good sensitivity of 94% and a notably lower specificity of 42%. Fisher’s exact test was applied to the confusion matrix (Table 1) and resulted in a *p* value < 0*.*001, indicating that the binary classifier performs better than random guessing and can indeed distinguish between rare diseases and common causes for chronic pain in the test setting. A receiver operating characteristic (ROC) curve was plotted and the R package pROC [41] was used to calculate the best threshold for classification of the given data set (Fig. 2, blue crosshair). This resulted in an optimal threshold for classification of 0.41, which led to a slightly lower sensitivity of 82%, but considerably increased specificity of 70%. The calculated 95% confidence band (light blue area) for sensitivity shows low variance for all thresholds. The area under the curve (AUC) of 0.82 indicates good separability between the four rare diseases and chronic pain.

**Table 1:** Confusion matrix RARE versus CP

		Predicted		
		RARE	CP	Sum_*true*_
True	RARE	200 (94%)	12 (6%)	212 (100%)
	CP	29 (58%)	21 (42%)	50 (100%)
	Sum_*predicted*_	228 (87%)	33 (13%)	261 (100%)

Percentages are relative to Sum_*true*_

##### The binary classifier of Pain2D can separate each of the four rare diseases from chronic pain with high sensitivity but low values for specificity

In addition to a general prediction of the presence of a rare disease vs. a common cause of chronic pain (Table 1, Fig. 2), we wanted also to test if the binary classifier of Pain2D is able to separate PDs of each of the four tested rare diseases (EDS, GBS, FSHD, PROMM) from PDs of more common causes of chronic pain (CP).

In these four cases, Pain2D classified PDs with an accuracy of ≥ 61% (Table 2). The most accurate classifier was for GBS versus CP (77%) and the most inaccurate one for FSHD versus CP (61%). Overall, the sensitivity achieved by the binary classifier of Pain2D for these four cases was ≥ 90%, with the best result for EDS versus CP at 98%. The values for specificity were relatively low at ≥ 30% (best result for GBS vs. CP at 66%).

**Table 2:** Classification results with the binary classifier of Pain2D for each rare disease versus CP, listing values for true positives (TP), false positives (FP), true negatives (TN), false negatives (FN), *p* value (Fisher’s exact test), accuracy (Acc), sensitivity (Sens), specificity (Spec), AUC of the ROC curve (AUC_ROC_)

	TP	FP	FN	TN	*p* value	Acc	Sens	Spec	AUC_ROC_
EDS versus CP	58	35	1	15	< 0*.*001	0.67	0.98	0.30	0.899 (CI 0.892–0.954)
GBS versus CP	28	17	1	33	< 0*.*001	0.77	0.96	0.66	0.921 (CI 0.853–0.973)
FSHD versus CP	34	32	1	18	< 0*.*001	0.61	0.97	0.36	0.854 (CI 0.77–0.93)
PROMM versus CP	80	24	9	26	< 0*.*001	0.76	0.90	0.52	0.846 (CI 0.774–0.908)

In all cases, the *p* values (Fisher’s exact test) suggest strongly that the classifier performs much better than random guessing

**Fig. 3:**
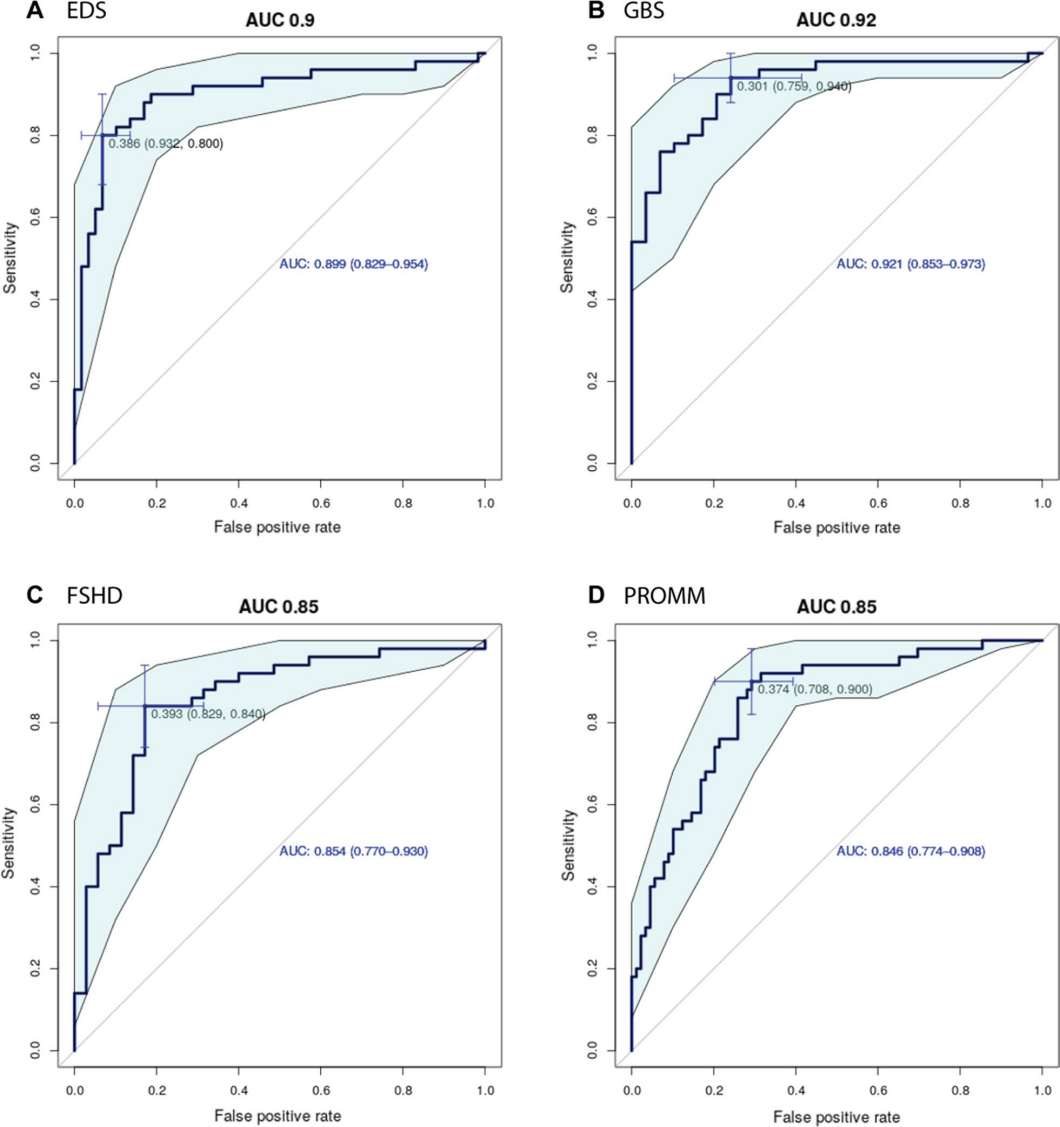
Receiver operating characteristics (ROC) curves for binary classification of each RD versus CP. **A** ROC curve binary classification of EDS and CP. AUC = 0.899 (CI 0.829–0.954), **B** ROC curve binary classification of GBS and CP. AUC = 0.921 (CI 0.853–0.973), **C** ROC curve binary classification of FSHD or CP. AUC = 0.854 (CI 0.770–0.930), **D** ROC curve binary classification of PROMM and CP. AUC = 0.846 (CI 0.774–0.908). Confidence intervals are depicted as light blue band. Blue crosshairs indicate optimal threshold for classification

AUCs of ROC curves were ≥ 0.845, with best results for GBS versus CP at 0.921 (Table 2, Fig. 3). Taken together, these results suggest that the binary classifier can distinguish between the control group and each of the four rare diseases investigated with good sensitivity, but considerably lower specificity.

##### The k-disease classifier of Pain2D can classify PDs as PROMM, EDS, FSHD, GBS and chronic pain with varying sensitivity and overall high specificity

The classification of all PDs with the k-disease classifier of Pain2D gave results with varying sensitivities for the five groups, ranging from 0.51 to 0.83 for the four diseases analyzed. The control group of unspecific chronic pain (CP) was classified with a low sensitivity of only 0.14. Specificity was overall high with values between 0.83 and 0.99 for the five diagnostic groups. In all cases, low *p* values of < 0*.*001 (*χ*^2^-Test) indicated that the k-diseases classifier performed better than random assignment of the diseases.

### Pain2D k-disease classification works well for EDS, GBS and FSHD

The classification for GBS, EDS and FSHD gave good results with sensitivities of 86%, 64% and 63%, respectively. Of note, EDS and GBS were previously efficiently classified with the binary classifier of Pain2D as well (sensitivity 86%, specificity 96%; [24]). As a possible explanation, the pain profiles of EDS and GBS each show painful regions that are not present in the other pain profiles, like the knees in EDS and the feet in GBS. These regions are only rarely marked in the other disease patterns, which could contribute to efficient classification. FSHD typically affects the shoulder and the lower back. These regions are also sometimes marked in PDs of other diseases, as well as in the unspecific chronic pain group. Nevertheless, the k-disease classifier of Pain2D was still able to classify FSHD with a sensitivity of 63% and specificity of 83%.

### Pain2D k-disease classification of PROMM PDs is less efficient

PROMM PDs were classified correctly only in 52%; the most common misclassifications were as GBS (23%) and FSHD (19%). Less efficient classification of PROMM could be the result of a number of reasons. The most likely explanation is based on a PD sample that is more heterogeneous than for the other diagnostic groups, for example due to the presence of different stages of the disease in the sample, with earlier stages differing with regard to the pain pattern from later stages. To address this, we indeed plan to perform a longitudinal analysis of the pain drawings in different disease stages as a follow up study.

The k-disease classifier of Pain2D achieved a high specificity for PROMM: only 17 non-PROMM PDs were classified as PROMM (false positives), resulting in a specificity of 90%. It is however impossible to say if this could be attributed to the higher sensitivities for classification of the other rare diseases as a result of their unique features in the pain profile (e.g., painful feet for GBS, etc.), or vice versa, or (most likely) both. Of note, PROMM was the biggest sample with 89 PDs in total, as opposed to only between 29 and 59 PDs for the other diagnostic groups.

### PDs from people with chronic pain are preferentially classified as any rare disease (EDS, GBS, FSHD, PROMM)

One reason why we included PDs from people with unspecific chronic pain in our study was to test if additional, non-specific pain patterns in patients with rare diseases (a “background pain noise”) changed the pain profile enough to interfere with classification. It turned out that the separation between CP and RD PDs was not an issue in our sample, as the number of false positives for the CP group was quite low with only 3 out of 211 rare disease PDs classified as CP (Table 3). In addition, the PDs of the CP diagnostic group were not preferentially classified as one specific rare disease, but were more or less randomly distributed into the five diagnostic groups (classification as PROMM in 14%, EDS in 26%, FSHD in 26% and GBS in 20%), which is reflected in the low sensitivity of 14% for CP (Table 3). Taken together, PD classification by Pain2D was not hampered in a relevant manner in our sample group by the putative presence of a background pattern in RD pain profiles.

**Table 3:** Confusion matrix resulting from classification of all PDs with the k-disease classifier of Pain2D

		Predicted					
		EDS	GBS	FSHD	PROMM	CP	Sum_true_
True	EDS	38	11	8	1	1	59
	GBS	1	25	0	3	0	29
	FSHD	4	3	22	5	1	35
	PROMM	5	20	17	46	1	89
	CP	13	10	13	7	7	50
Sum_predicted_		61	69	60	62	10	262
Sensitivity		0.644	0.862	0.629	0.517	0.140	
Specificity		0.887	0.811	0.833	0.908	0.986	

## Discussion

### Comparison of Pain2D generated pain profiles (PPs) to pain patterns described in the literature

We were able to show that Pain2D is a useful tool to generate disease specific pain patterns and utilize them for automated diagnostic support. The pain pattern of FSHD shows a similar distribution as described by Morís et al. [42]. The percentages they observed are slightly lower (72% as opposed to up to 90% for backpain), but the main localizations are the same. As expected, the Pain2D PP shows higher resolution (e.g., high percentages marked specifically the knees, while Morís et al. report only general leg pain). The Pain2D generated EDS PP correlates with the description of EDS in the literature, as joint and spinal pain are known typical manifestations of EDS [43].

Similar observations can be made for GBS. Pain in distal extremities is a common symptom in GBS described in the literature [44], which fits to the Pain2D generated PP for GBS (~ 70% marked the feet). Of note, the Pain2D PP shows that the plantar sides of the feet are marked by far more patients than the dorsal side or the distal leg, and thereby adds details to the currently described observations with regard to pain in GBS.

Classification of PROMM PDs was less efficient than for the other diagnostic groups (52%), which seems to be related to more heterogeneous PDs. It is worth mentioning that the PROMM PDs differed with regard to the presence or absence of pain in the upper legs, which might be one factor that hampers classification. Indeed, descriptions of the pain pattern of PROMM vary a lot in the literature. The Pain2D generated PROMM pain profile generally fits to the findings of Eger et al. [45]. In contrast, Peric et al. [46] observed higher pain frequencies in the lower legs (41%) than in the upper legs (30%) compared to the Pain2D PP (~ 20 and ~ 60%, respectively). Our results show a higher difference in the frequencies between upper and lower legs compared to prior published results, and we obtained overall lower pain frequencies in nearly all body regions compared to George et al. (e.g., 80% in upper legs) [47]. Taken together, the pain pattern in PROMM seems to differ among patients, which is consistent with more difficult classification. Indeed, its variable clinical manifestation is often discussed as a reason for PROMM being an underdiagnosed disease [39].

### Potential usefulness of Pain2D as a DDSS

The binary classifier of Pain2D achieved a sensitivity of over 90% for all the diseases we investigated versus chronic pain, suggesting that Pain2D might be a useful tool to ensure that rare diseases are taken into consideration for the differential diagnosis of unclear pain manifestations. Furthermore, since the binary classifier performed generally with high accuracy, it might be useful in the differential diagnosis of two similar diseases.

Generally speaking, many rare diseases present with pain as one of the first symptoms prompting patients to see a physician. For example, pain is described as one of the first symptoms of FSHD [33]. For PROMM, a study of 2013 has shown that leg pain is the first symptom for the disease in 5.2% of cases, in addition to a similar fraction that presents with general pain at first [48]. As both pain types could potentially be detected by PD, we hope that Pain2D can contribute to the process of diagnosis for these patients in the future. In addition, all Pain2D applications are published as open-source and can be trained to generate specific pain profiles for many, if not most, rare diseases manifesting with pain as a symptom.

The sensitivity of the k-disease classifier of Pain2D ranged between 52 and 86% for our test sample. FSHD classification achieved a sensitivity of 63% and a specificity of 83%, compared to molecular genetic testing for FSHD with a sensitivity of 93% and a specificity of ca. 98% [49]. As discussed above, PROMM was classified with a rather low sensitivity of 52% and specificity of 91%. GBS was classified best with a sensitivity of 86% and a specificity of 81%. Standard diagnosis of GBS is based on clinical features, electrophysiological studies and cerebrospinal fluid analysis [29, 30], so that no numerical results can be given for comparison. EDS classification achieved a sensitivity of 64% and specificity of 89%. Since EDS comprises a number of different entities, genetic testing is only available for some of those, while the most prevalent form, the hypermobile EDS, is diagnosed based solely on clinical evaluation with unknown accuracy. Given the simplicity and non-invasive nature of generating a pain drawing, a fully trained version of Pain2D with a comprehensive library of pain profiles could therefore indeed efficiently guide physicians in the search for rare diseases by pointing out the more likely candidates in order to initiate specific diagnostic procedures, especially when pain drawings are enhanced with additional patient information, for example from questionnaires. It thus could contribute to shortening the “diagnostic odyssey” that patients with rare diseases often have to endure. In specific cases, it might even develop as a diagnostic indicator in its own right, conceivably in the differentiation of two similar diseases with a clear distinction in pain pattern.

### Challenges and future directions

Although Pain2D achieved overall good results with classification of the five tested diagnostic groups, there are some limitations to the tool as of now. Firstly, the chosen classification strategy is sensitive to the presence of more than one pain pattern in one disease, which could be the result of, e.g., changes over time and progression of the disease. This is due to the fact that similarity is measured against the PP, which could be understood as an “average PD” of the disease, that potentially masks the presence of more than one pattern per disease. Diseases with more than one “typical” manifestation of pain, or the presence of more complex pain patterns, could therefore pose a problem for Pain2D. For example, k-disease classification of PROMM PDs turned out to be less sensitive then for the other groups (sensitivity 52%, specificity 91%), in part due to the presence of at least two subgroups of pain patterns in the PP (thighs marked vs. thighs not marked). While the chosen approach (similarity to PP) achieved superior results compared to a nearest neighbor classifier, which was tested during Pain2D development, this issue has to be addressed in the future. As one likely reason for the presence of different disease patterns in patients with the same disease are changes over time, a longitudinal study to follow disease progression is currently in preparation. In this case, as well as other cases of “more than one pain pattern” per disease, training Pain2D with subgroups could overcome the problem, as long as enough PDs per subgroup are available for training.

Secondly, for now Pain2D can recognize only four rare diseases and is based on a limited number of labeled data. This could be remedied over time by collecting more PDs. However, acquiring enough training data for a rare disease classifier is generally a challenge. Not only is the number of people suffering from a specific rare disease limited, but, as of now, ~ 7000 rare diseases are known, and the number is still growing. Pain2D therefore needs to be trained for many RDs presenting with pain before it can become a generally useful DDSS. PDs of many RDs need to be sampled and compiled into PPs, which is almost impossible for some ultra-rare diseases with only a few known cases. While this is a general unresolved problem for DDSS dealing with rare or ultra-rare diseases, there are attempts to overcome it. For example, the facial phenotype analyzer Face2Gene [12, 13] has recently been complemented by the GestaltMatcher algorithm, an encoder for portraits that can match patients with similar disorders that were not included in the training data. It accomplishes this by placing individual portraits in a Clinical Face Phenotype Space derived from the training data, in which distances between cases represent syndromic similarity [14]. While this is a rather elegant approach, it is restricted to deep learning methods and needs a lot of training data, which we currently don’t have. Nevertheless, Pain2D can potentially grow over time by collecting data from many researchers/physicians, as it is available as a free and open-source tool.

Lastly, while classification of PDs with Pain2D-Tool worked overall well with our test data set, one has to take into account that the latter is not a representative sample of patients suffering from pain without a diagnosis. Prevalences of rare diseases are by definition low, with consequences for classification results in a clinical setting. For example, the k-disease classifier preferentially mis-classified PDs from the CP group as rare (43 of 50, 86%), although the latter are far more often encountered in practice. As a result, many PDs in a realistic setting would likely be classified as rare (of which only a small percentage is truly rare), and the positive predictive value would be rather low due to its specificity and low prevalence. However, in the case of rare diseases, higher false positive rates/lower specificities are less problematic, as RDs are typically highly underdiagnosed, not overdiagnosed. As diagnostic aids for RDs are targeted towards helping physicians to take rare differential diagnoses into account, they are not meant as diagnostic methods on their own. Final diagnosis has always to be confirmed with other diagnostic procedures, making lower specificities more tolerable. Nevertheless, overall accuracy could potentially be improved by pre-testing in order to triage patients likely suffering from an undiagnosed rare disease. This could for example be achieved by using Pain2D in combination with the Q53 questionnaire, which classifies answer patterns into rare somatic, common somatic, and psychosomatic diseases with sensitivities between 87 and 89% and specificities between 84 and 88% [11]. Taken together, as Pain2D is a non-invasive and cost-efficient software, its strength is the suggestion of possible diagnoses for further testing in order to abbreviate the diagnostic odyssey patients with rare diseases often have to endure, and we consider higher numbers of false positives from Pain2D to be an acceptable intermediate step towards this goal.

Compared to our initial results with Pain2D [24], this study has two important innovations. Firstly, we were able to show that Pain2D is indeed able to distinguish between two related diseases of the same category (neuromuscular disorders; PROMM and FSHD). Secondly, we successfully used the new k-disease classifier of Pain2D for the classification of five diagnostic groups, including four rare diseases.

In conclusion, our study could show that Pain2D has the potential to develop into a full DDSS for pain-associated diseases with a focus on rare diseases, and opens up the route towards further exploitation of pain symptoms for AI-assisted diagnosis of rare diseases.

## Data Availability

All data discussed are included with the published article.

## References

[1] About Rare Diseases | www.eurordis.org. https://www.eurordis.org/about-rare-diseases. Accessed 9 Feb 2022.

[2] FAQs About Rare Diseases | Genetic and Rare Diseases Information Center (GARD)—an NCATS Program. https://rarediseases.info.nih.gov/diseases/pages/31/faqs-about-rare-diseases. Accessed 9 Feb 2022.

[3] Stieber C, Mücke M, Windheuser IC, Grigull L, Klawonn F, Tunc S, Münchau A, Klockgether T (2017). On the fast track to diagnosis: recommendations for patients without a diagnosis. Bundesgesundheitsblatt Gesundheitsforschung Gesundheitsschutz.

[4] Schaefer J, Lehne M, Schepers J, Prasser F, Thun S (2020). The use of machine learning in rare diseases: a scoping review. Orphanet J Rare Dis.

[5] Faviez C, Chen X, Garcelon N, Neuraz A, Knebelmann B, Salomon R, Lyonnet S, Saunier S, Burgun A (2020). Diagnosis support systems for rare diseases: a scoping review. Orphanet J Rare Dis.

[6] Schaaf J, Sedlmayr M, Schaefer J, Storf H (2020). Diagnosis of rare diseases: a scoping review of clinical decision support systems. Orphanet J Rare Dis.

[7] Sutton RT, Pincock D, Baumgart DC, Sadowski DC, Fedorak RN, Kroeker KI (2020). An overview of clinical decision support systems: benefits, risks, and strategies for success. NPJ Digit Med.

[8] Berner ES (2006). Diagnostic decision support systems: why aren’t they used more and what can we do about it?. AMIA Annu Symp Proc.

[9] Grigull L, Lechner W, Petri S (2016). Diagnostic support for selected neuromuscular diseases using answer-pattern recognition and data mining techniques: a proof of concept multicenter prospective trial. BMC Med Inform Decis Mak.

[10] Mücke U, Klemann C, Baumann U, Meyer-Bahlburg A, Kortum X, Klawonn F, Lechner WM, Grigull L (2017). Patient’s experience in pediatric primary immunodeficiency disorders: computerized classification of questionnaires. Front Immunol.

[11] Grigull L, Mehmecke S, Rother A-K, Blöß S, Klemann C, Schumacher U, Mücke U, Kortum X, Lechner W, Klawonn F (2019). Common pre-diagnostic features in individuals with different rare diseases represent a key for diagnostic support with computerized pattern recognition?. PLoS ONE.

[12] Gurovich Y, Hanani Y, Bar O (2019). Identifying facial phenotypes of genetic disorders using deep learning. Nat Med.

[13] Hsieh T-C, Mensah MA, Pantel JT (2019). PEDIA: prioritization of exome data by image analysis. Genet Med.

[14] Hsieh T-C, Bar-Haim A, Moosa S (2022). GestaltMatcher facilitates rare disease matching using facial phenotype descriptors. Nat Genet.

[15] Subirats L, Reguera N, Bañón AM, Gómez-Zúñiga B, Minguillón J, Armayones M (2018). Mining facebook data of people with rare diseases: a content-based and temporal analysis. Int J Environ Res Public Health.

[16] Palmer H (1949). Pain charts; a description of a technique whereby functional pain may be diagnosed from organic pain. N Z Med J.

[17] Shaballout N, Neubert T-A, Boudreau S, Beissner F (2019). From paper to digital applications of the pain drawing: systematic review of methodological milestones. JMIR Mhealth Uhealth.

[18] Egloff N, Gander M, Cámara R, Klingler N, Wegmann B, Marti E, von Känel R (2011). Pain drawings help to distinguish between somatic and somatoform pain. PPmP - Psychotherapie · Psychosomatik · Medizinische Psychologie.

[19] Mann NH, Brown MD (1991). Artificial intelligence in the diagnosis of low back pain. Orthop Clin N Am.

[20] Hüllemann P, Keller T, Kabelitz M, Freynhagen R, Tölle T, Baron R (2017). Pain drawings improve subgrouping of low back pain patients. Pain Pract.

[21] Tachibana T, Maruo K, Inoue S, Arizumi F, Kusuyama K, Yoshiya S (2016). Use of pain drawing as an assessment tool of sciatica for patients with single level lumbar disc herniation. Springerplus.

[22] Rennerfelt K, Zhang Q, Karlsson J, Styf J (2018). Patient pain drawing is a valuable instrument in assessing the causes of exercise-induced leg pain. BMJ Open Sport Exerc Med.

[23] Bender TTA, Leyens J, Sellin J, Kravchenko D, Conrad R, Mücke M, Seidel MF (2020). Therapeutic options for patients with rare rheumatic diseases: a systematic review and meta-analysis. Orphanet J Rare Dis.

[24] Wester L, Mücke M, Bender TTA, Sellin J, Klawonn F, Conrad R, Szczypien N (2020). Pain drawings as a diagnostic tool for the differentiation between two pain-associated rare diseases (Ehlers–Danlos-syndrome, Guillain–Barré-syndrome). Orphanet J Rare Dis.

[25] Zhou Z, Rewari A, Shanthanna H (2018). Management of chronic pain in Ehlers–Danlos syndrome: two case reports and a review of literature. Medicine (Baltimore).

[26] Germain D-P (2017). Ehlers-Danlos syndromes. Ann Dermatol Venereol.

[27] Peña L, Moreno CB, Gutierrez-Alvarez AM (2015). Pain management in Guillain–Barre syndrome: a systematic review. Neurología (English Edition).

[28] Fujimura H (2013). The Guillain–Barré syndrome. Handb Clin Neurol.

[29] Leonhard SE, Mandarakas MR, Gondim FAA (2019). Diagnosis and management of Guillain–Barré syndrome in ten steps. Nat Rev Neurol.

[30] Liu S, Dong C, Ubogu EE (2018). Immunotherapy of Guillain–Barré syndrome. Hum Vaccin Immunother.

[31] Statland J, Tawil R (2014). Facioscapulohumeral muscular dystrophy. Neurol Clin.

[32] Deenen JCW, Arnts H, van der Maarel SM, Padberg GW, Verschuuren JJGM, Bakker E, Weinreich SS, Verbeek ALM, van Engelen BGM (2014). Population-based incidence and prevalence of facioscapulohumeral dystrophy. Neurology.

[33] Bushby KMD, Pollitt C, Johnson MA, Rogers MT, Chinnery PF (1998). Muscle pain as a prominent feature of facioscapulohumeral muscular dystrophy (FSHD): four illustrative case reports. Neuromuscul Disord.

[34] Hamel J, Tawil R (2018). Facioscapulohumeral muscular dystrophy: update on pathogenesis and future treatments. Neurotherapeutics.

[35] Turner C, Hilton-Jones D (2010). The myotonic dystrophies: diagnosis and management. J Neurol Neurosurg Psychiatry.

[36] Mahyera AS, Schneider T, Halliger-Keller B, Schrooten K, Hörner E-M, Rost S, Kress W (2018). Distribution and structure of DM2 repeat tract alleles in the German population. Front Neurol.

[37] Vanacore N, Rastelli E, Antonini G (2016). An age-standardized prevalence estimate and a sex and age distribution of myotonic dystrophy types 1 and 2 in the Rome Province, Italy. Neuroepidemiology.

[38] Suominen T, Bachinski LL, Auvinen S, Hackman P, Baggerly KA, Angelini C, Peltonen L, Krahe R, Udd B (2011). Population frequency of myotonic dystrophy: higher than expected frequency of myotonic dystrophy type 2 (DM2) mutation in Finland. Eur J Hum Genet.

[39] Meola G, Cardani R (2017). Myotonic dystrophy type 2 and modifier genes: an update on clinical and pathomolecular aspects. Neurol Sci.

[40] Suokas KI, Haanpää M, Kautiainen H, Udd B, Hietaharju AJ (2012). Pain in patients with myotonic dystrophy type 2: a postal survey in finland. Muscle Nerve.

[41] Robin X, Turck N, Hainard A, Tiberti N, Lisacek F, Sanchez J-C, Müller M (2011). pROC: an open-source package for R and S+ to analyze and compare ROC curves. BMC Bioinform.

[42] Morís G, Wood L, FernáNdez-Torrón R (2018). Chronic pain has a strong impact on quality of life in facioscapulohumeral muscular dystrophy. Muscle Nerve.

[43] Gazit Y, Jacob G, Grahame R (2016). Ehlers–Danlos syndrome-hypermobility type: a much neglected multisystemic disorder. Rambam Maimonides Med J.

[44] Moulin DE, Hagen N, Feasby TE, Amireh R, Hahn A (1997). Pain in Guillain–Barré syndrome. Neurology.

[45] Eger K, Schulte-Mattler WJ, Zierz S (1997). Proximale myotone Myopathie (PROMM)Klinische Variabilität innerhalb einer Familie. Nervenarzt.

[46] Peric M, Peric S, Rapajic N, Dobricic V, Savic-Pavicevic D, Nesic I, Radojicic S, Novakovic I, Lavrnic D, Rakocevic-Stojanovic V (2015). Multidimensional aspects of pain in myotonic dystrophies. Acta Myol.

[47] George A, Schneider-Gold C, Zier S, Reiners K, Sommer C (2004). Musculoskeletal pain in patients with myotonic dystrophy type 2. Arch Neurol.

[48] Hilbert JE, Ashizawa T, Day JW, Luebbe EA, Martens WB, McDermott MP, Tawil R, Thornton CA, Moxley RT (2013). Diagnostic odyssey of patients with myotonic dystrophy. J Neurol.

[49] Tawil R, Kissel JT, Heatwole C, Pandya S, Gronseth G, Benatar M (2015). Evidence-based guideline summary: evaluation, diagnosis, and management of facioscapulohumeral muscular dystrophy. Neurology.

